# Crops *In Silico*: Generating Virtual Crops Using an Integrative and Multi-scale Modeling Platform

**DOI:** 10.3389/fpls.2017.00786

**Published:** 2017-05-15

**Authors:** Amy Marshall-Colon, Stephen P. Long, Douglas K. Allen, Gabrielle Allen, Daniel A. Beard, Bedrich Benes, Susanne von Caemmerer, A. J. Christensen, Donna J. Cox, John C. Hart, Peter M. Hirst, Kavya Kannan, Daniel S. Katz, Jonathan P. Lynch, Andrew J. Millar, Balaji Panneerselvam, Nathan D. Price, Przemyslaw Prusinkiewicz, David Raila, Rachel G. Shekar, Stuti Shrivastava, Diwakar Shukla, Venkatraman Srinivasan, Mark Stitt, Matthew J. Turk, Eberhard O. Voit, Yu Wang, Xinyou Yin, Xin-Guang Zhu

**Affiliations:** ^1^Department of Plant Biology, University of Illinois at Urbana–Champaign, UrbanaIL, USA; ^2^Carl R. Woese Institute for Genomic Biology, University of Illinois, Urbana–Champaign, UrbanaIL, USA; ^3^Department of Crop Sciences, University of Illinois, UrbanaIL, USA; ^4^United States Department of Agriculture – Agricultural Research Service–Donald Danforth Plant Science Center, St. LouisMO, USA; ^5^Department of Astronomy–College of Education, University of Illinois at Urbana–Champaign, UrbanaIL, USA; ^6^Department of Molecular & Integrative Physiology, University of Michigan, Ann ArborMI, USA; ^7^Department of Computer Graphics Technology, Purdue University, West LafayetteIN, USA; ^8^ARC Centre of Excellence for Translational Photosynthesis, Research School of Biological Sciences, Australian National University, ActonACT, Australia; ^9^National Center for Supercomputing Applications, University of Illinois at Urbana–Champaign, UrbanaIL, USA; ^10^Department of Computer Science, University of Illinois at Urbana–Champaign, UrbanaIL, USA; ^11^Department of Horticulture and Landscape Architecture, Purdue University, West LafayetteIN, USA; ^12^Department of Plant Science, Pennsylvania State University, University ParkPA, USA; ^13^Centre for Plant Integrative Biology, University of NottinghamNottingham, UK; ^14^SynthSys and School of Biological Sciences, Edinburgh UniversityEdinburgh, UK; ^15^Department of Chemical and Biomolecular Engineering, University of Illinois at Urbana–Champaign, UrbanaIL, USA; ^16^Institute for Systems Biology, SeattleWA, USA; ^17^Department of Computer Science, University of Calgary, CalgaryAB, Canada; ^18^Max Planck Institute of Molecular Plant PhysiologyGolm, Germany; ^19^School of Information Science, University of Illinois, Urbana–Champaign, UrbanaIL, USA; ^20^The Wallace H. Coulter Department of Biomedical Engineering, Georgia Tech and Emory University, AtlantaGA, USA; ^21^Centre for Crop Systems Analysis, Department of Plant Sciences, Wageningen University & ResearchWageningen, Netherlands; ^22^CAS Key Laboratory for Computational Biology–State Key Laboratory for Hybrid Rice, Partner Institute for Computational Biology, Chinese Academy of SciencesShanghai, China

**Keywords:** crop yield, multiscale, computational framework, model, integration

## Abstract

Multi-scale models can facilitate whole plant simulations by linking gene networks, protein synthesis, metabolic pathways, physiology, and growth. Whole plant models can be further integrated with ecosystem, weather, and climate models to predict how various interactions respond to environmental perturbations. These models have the potential to fill in missing mechanistic details and generate new hypotheses to prioritize directed engineering efforts. Outcomes will potentially accelerate improvement of crop yield, sustainability, and increase future food security. It is time for a paradigm shift in plant modeling, from largely isolated efforts to a connected community that takes advantage of advances in high performance computing and mechanistic understanding of plant processes. Tools for guiding future crop breeding and engineering, understanding the implications of discoveries at the molecular level for whole plant behavior, and improved prediction of plant and ecosystem responses to the environment are urgently needed. The purpose of this perspective is to introduce Crops *in silico* (cropsinsilico.org), an integrative and multi-scale modeling platform, as one solution that combines isolated modeling efforts toward the generation of virtual crops, which is open and accessible to the entire plant biology community. The major challenges involved both in the development and deployment of a shared, multi-scale modeling platform, which are summarized in this prospectus, were recently identified during the first Crops *in silico* Symposium and Workshop.

## Introduction

Designing crops with higher yield potential and enhanced resource use efficiency is a desirable goal for future food security and sustainability. However, this is a difficult task for crop breeding and engineering programs due to unforeseen, complex traits that arise from interactions among genotype, environment, and management (GxExM). Tools that predict emergent phenotypes in response to genetic or environmental perturbations by identifying metabolic pathways or canopy forms for modification are needed to evaluate and ameliorate risks to crop yield (Yin and Struik, 2008; Srinivasan et al., 2017). The information obtained from these tools can be used to direct breeding efforts to design new germplasm (ideotypes) that can thrive in a variety of environmental scenarios.

The accurate reconstruction of organisms *in silico* is a timely solution toward increasing our predictive capabilities. This prospect has been at the forefront of vertebrate and microbial modeling efforts for the last two decades. Many successes have been realized from community projects based on integrative, multi-scale modeling built around a central framework and supported by their respective research communities, such as the Virtual Physiological Human (VPH) (Hunter et al., 2013), the Virtual Physiological Rat (VPR) (Beard et al., 2012), and in a whole cell model of *Mycoplasma genitalium* (Karr et al., 2012). The VPH and VPR projects have made significant strides toward the realization of predictive medicine via working examples of integrative and multi-scale modeling (Tewari et al., 2016).

Many robust models have been developed to simulate biological processes and phenotypic responses of crops to environmental parameters, including models of: the C3 and C4 photosynthetic process (Zhu et al., 2013; Wang et al., 2014); 3D plant canopies (Song et al., 2013); stomatal action (Buckley and Mott, 2013); respiration (Sweetlove et al., 2013); phloem and xylem flow (Hall and Minchin, 2013); growth and development (Prusinkiewicz and Runions, 2012); flowering (Song et al., 2012); root structural and functional dynamics (Lynch, 2013); and gene regulatory networks (Chew et al., 2014, 2017), among others. However, many isolated crop models focus on a narrow range of spatial and temporal scales, limiting their ability for extrapolation beyond measured data and resulting in inadequate prediction of crop response to new scenarios produced by perturbations (Zhu et al., 2016). There is a need to rebuild crop growth models to include the underlying mechanisms of response, reaching from gene networks and metabolic pathways through to cellular organization, tissue and organ development, and resource capture in dynamic competitive environments and ecosystems.

Despite the rich history of robust plant systems modeling (Tardieu, 2010), no coordinated effort toward the creation of a virtual physiological plant, based on integrative and multi-scale modeling, has been initiated or sustained in the plant community. While the mammalian, microbial, earth systems, hydrological, and astrophysical communities, among others, have developed methods and tools to overcome many obstacles in integrative and multi-scale modeling, and which can be adapted toward modeling plant growth, several challenges are unique to the plant community.

Some specific challenges to integrative and multi-scale modeling in plants were identified by the international community of scientists at the first symposium and workshop on Plants *in silico* (now renamed as Crops *in silico*). First, while there is only one species of human, there are dozens of food and bioenergy crops in production with important differences in primary and secondary metabolism, plant architecture, and phenology, which require different models for accurate simulations. Second, isolated modeling efforts have resulted in redundancy and a collection of models written in different markup and scripting languages due to a lack of community standards. Code and documentation for many legacy models are either difficult to find or are publicly unavailable, while many new models lack a user interface or meaningful data visualization. Another technical barrier is integrating models at different spatial and temporal scales, while social barriers include issues of intellectual property and ownership of code and model inputs/outputs. High level plant models are often inaccessible to the research community that does not have computational expertise. Many of these models could be improved with better estimates of model parameters supplied by domain experts.

To overcome these limitations, this first workshop discussed the following aims and goals. The long-term aim of Crops *in silico* is to reconstruct a functioning crop plant and community of plants from the genes upward. A secondary, but equally important, aim is to transition crop and plant modeling from many siloed efforts to a whole community effort that can benefit from the synergies that are largely absent today. Toward this aim the following goals must be fulfilled. (1) Provide a framework that enables integration of models at different levels from gene and metabolic networks to organ development and whole crop productivity. (2) Develop the framework to be crop independent, to avoid recreating common processes for each crop, such as photosynthesis (i.e., crop specification in parameter files rather than hard-wired into the code). (3) Plug-and-play capability for fine- or coarse-graining biological processes. (4) Provide a user-friendly graphical interface to facilitate use by domain experts. (5) Deliver outputs as 3D visualizations and animations. To achieve these goals, the plant sciences community must develop a close partnership with computer science to achieve a joint mission.

## The Crops *In Silico* Initiative

**FIGURE 1:**
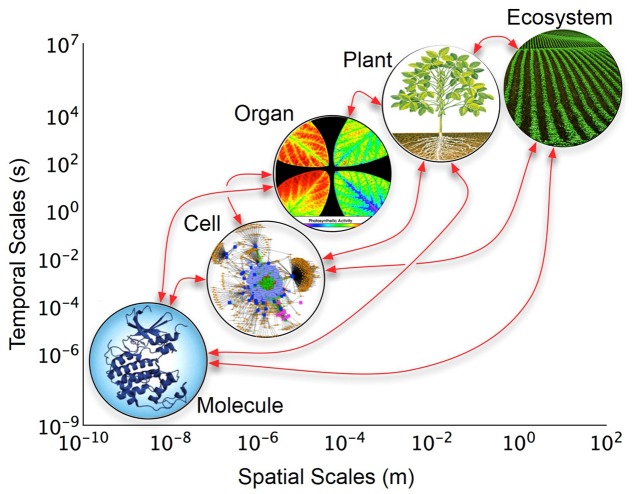
**Layers of organization of biological models across temporal and spatial scales.** The *y*-axis represents real-time in which changes occur at each biological level; the *x*-axis represents the relative size or space which the biological level encompasses. The arrows indicate possible direct interactions among scales. Organ level image is from Kim et al. (2001).

We propose to meet the above stated challenges and goals through the creation of a discovery platform called Crops *in silico* (Cis)^1^. Developments in high-performance computing (HPC), open-source software, and functional knowledge of plants render the Cis concept realistic and timely. Crops *in silico* is envisioned as a central framework of tools and modules that can be interconnected to solve user-defined biological questions. With a large enough collection of tools and modules we can achieve an accurate representation of a reference plant (*Arabidopsis thaliana*) as well as individuals and fields of crop plants such as rice (*Oryza sativa*), maize (*Zea mays*), soybean (*Glycine max*), and cassava (*Manihot esculenta*), spanning from the fine-grained atomistic scale, up to the coarse-grained whole plant or ecosystem scale (**Figure 1**). A suggested roadmap for future directions was developed by the participants of the symposium, which is outlined here, and includes: (i) building a community of researchers from different domains of expertise; (ii) construction of a central framework to build the virtual crops; (iii) incorporation of existing, or development of new resources; and (iv) continual improvement of model integration and mathematical descriptions of underlying natural processes.

## Roadmap

### The Research Community

To achieve transformational advances in the development of virtual crops, it is essential to build an international Crops *in silico* community comprised of experts in experimentation, agronomy, physiology, phenotyping, breeding, modeling, computer science, software development, and visualization. It is crucial for a community of domain experts to come together in collaborations and conversations to understand diverse needs, best estimates of parameters, and ongoing biological and technical questions to drive the development of the Cis framework. A Cis community has the capacity to share models and unique data sets, providing the information needed to form more complete crop models with more accurate data.

As a community we can address many of the issues that commonly plague integrative and multi-scale modeling efforts in plants. Some examples include: the adoption of a common lexicon, sustainable data and model standards to facilitate the design, implementation of software tools that enable model interoperability, and agreed rules on sharing and archiving of model components. Others in the biological modeling community have addressed these issues by establishing organizations such as the Wheat Data Interoperability Working Group^2^, or the Computational Modeling in Biology Network (COMBINE)^3^. Similarly, the Cis community must adopt and support standards and network with the larger computational biology neighborhood to take full advantage of open resources for model interoperability.

To enable this communication, annual meetings will facilitate collaboration among plant scientists and the larger modeling community in two forums. The annual science symposium and workshops will share research from isolated efforts and organize collaborative thinking toward generating virtual crops. The symposium will comprise presentations on recent advancements in modeling plants at different levels of organization and computational tools that could accelerate achieving *in silico* plants. During the workshop, participants will (i) discuss the latest developments within and the linkages across science domains, (ii) share new methods and resources during hands-on tutorials for users, and (iii) develop strategic plans to advance the Cis project; specifically realizing the integrated framework. A separate hackathon event will develop a community of software tool developers and infrastructure resource providers, to collaborate on standards development and software support, including software integration.

### The Central Framework

Critical to the success of existing multi-scale modeling efforts in other fields has been the establishment of a centralized framework capable of connecting, integrating, and running models. A modeling infrastructure facilitates sharing of data and tools and has sufficient interface development to enable users at all levels of expertise to take advantage of plug-and-play modeling. Complex module integration will require collaboration among engineers, mathematicians, computer scientists, and biologists to develop a suite of pre-processing and processing resources to: (i) expedite semantic reconciliation among model languages; (ii) give new emphasis to model annotation using standardized ontologies and vocabularies; and (iii) perform biologically driven module reduction.

Many successful frameworks already exist that the Cis community can build upon and learn from. Within the plant community there is the Virtual Laboratory (VLab)/L-studio environment (Prusinkiewicz, 2004), which is widely used to model plant development at scales ranging from cells and tissues to individual plants to plant ecosystems using the integrative power of the L-system formalism^4^, and the OpenAlea (Pradal et al., 2008) framework, which takes a top-down approach and focuses on integrating ecophysiological and agronomic processes with plant architecture models. Cactus (Goodale et al., 2003) is one such open-source component framework from the astrophysics community, for HPC in which modularity allows components to be run at different scales for different applications. Similarly, the OpenMI interface (Moore and Tindall, 2005), enables multiscale hydrology modeling and provides adopted community standards and a framework to exchange data between environmental and water management models at runtime.

The Cancer, Heart and Soft Tissue Environment (CHASTE) supports biological multi-scale models, combining models of different types in a modular fashion for several common scenarios (49 publications since 2008^5^). As another example, the VPR project created a modeling framework called SemGen (Neal et al., 2015) that contains software allowing users to perform fine- and coarse-graining of aggregate models at the organ and cellular levels, where specific pathways can be extracted as modules. Whereas Cactus and OpenMI rely on developers to write models in a supported language, SemGen takes advantage of adopted community standards such as CellML^6^ and SBML^7^, and provides tools to annotate existing models with rich semantics when standards fail with certain model types (e.g., partial differential equations). For loosely coupled simulations, where many components interact strictly through file exchanges, the Swift framework (Wilde et al., 2011), which takes advantage of a variety of computational resources, might be appropriate. The proto-Crops *in silico* community includes researchers from the Cactus, VPR, and Swift projects who will advise on the construction of an appropriate framework for the plant science community.

### Integration of Models, Data, Tools, and Visualization

Long-term model integration will benefit greatly from coupling well-documented transmission standards with packaged communication libraries, which will alleviate common issues related to ensuring bit-level interoperability, and allow researchers to focus on semantic interoperability. Some of these existing resources are described below. The inclusion of information from many research groups, species, and environments will improve the quality of outputs and expand the utility of Cis to answer a diverse set of research questions. Existing software, tools, and visualization resources can be leveraged to create the modular framework capable of performing the necessary simulations. One initial method for model communication put forward in the workshop is the Advanced Message Queuing Protocol (AMQP^8^). This protocol is suitable for coarse-grained communication among models written in different languages (Python, MATLAB, C/C++, etc.) and operating at different time steps. MPI^9^ is an alternative messaging protocol for models requiring fine-grained communication, while the Swift^10^ approach was suggested to enable parallelism at different levels of granularity. We envision that the Cis framework will include a set of libraries that provide interoperability and communication between models, including the AMQP or MPI. The Cis platform will be the total environment, encompassing the user interface for viewing and launching models, libraries, data repositories, and so forth.

#### Model Repositories

Using the packaged Cis framework interoperability and communication libraries, models will be linked during execution (**Table 1**). BioModels (Chelliah et al., 2015) is a large model repository which provides access to published pathway models, and has automatic conversion tools that provide downloads in multiple formats. PlaSMo (Tindal et al., 2010) is a smaller and specific portal for plant growth models, which may be published or private. These range from general crop level models, such as LINTUL^11^, which simulates plant biomass accumulation based on light interception and efficiency in crops, to more specific plant processes, including models that cannot be represented in SBML. PlaSMo supports SBML models, including updated versions of models that are published in BioModels.

**Table 1:** Existing tools and resources for integrative and multi-scale modeling.

	Resource	Description	Citation
Frameworks	Cactus	Problem solving framework that enables parallel computation across scales.	Goodale et al., 2003
	SemGen	Tool to automate modular composition and decomposition of biosimulation models	Gennari et al., 2011; Neal et al., 2015
	FLAME	Agent-based modeling system that scales from laptops to HPC and parallel super computing	Holcombe et al., 2006; Kiran et al., 2010
	OpenMI	Software for independent model exchange at run time.	Moore and Tindall, 2005
	Swift	Parallel scripting system for many task workflows.	Wilde et al., 2011
	VLab/L-studio	Modeling and simulation of plant development from genes to ecosystems	Prusinkiewicz, 2004
	OpenAlea	Visualization and modeling of plant architecture.	Pradal et al., 2008
Model/data repositories	PlaSMo	Database for plant growth models and interface,	Tindal et al., 2010
	BioModels	Database with biochemical and non-biochemical models, MIRIAM compliant	Chelliah et al., 2015
	GEO	Data repository for high throughput genomic datasets, utilizing MIAME standards	Edgar et al., 2002
	CyVerse	Repository for tools for developing data storage pipeline	Hanlon et al., 2015; Merchant et al., 2016
Semantic reconciliation	SBOL	Standard synthetic biology open language	Roehner et al., 2016
	JSim	Utilizes mathematical modeling language for writing models and annotation	Butterworth et al., 2013
	COMBINE	Initiative to develop a set of interoperable and non-overlapping standards for modeling	Hucka et al., 2003

#### Data Repositories

The Cis framework will support access to existing experimental data repositories, such as GEO (Edgar et al., 2002), the AgMIP Data Interchange^12^, CyVerse (Merchant et al., 2016), KiMOSys (Costa et al., 2014), and BetyDB^13^. Importantly, Cis must be able to assess the origin and reliability of data used in the various modules to prevent the propagation of errors. This will require the community to gain a sustainable consensus on strategies and metrics for evaluating the credibility of both the input data and the integrated model outputs, including uncertainty quantification, sensitivity analysis, error documentation, version tracking, and validation with experimental data. Recognizing that the most influential models in plant sciences have resulted from close interaction between model and experiment, Cis should be designed to enable all interested labs to exploit the modeling framework in advancing their experimental and observational studies, in turn providing validation and improvements.

#### Software and Tools

The technical integration of models requires software to overcome the dual challenge of reconciling the semantics of models at different scales and successfully leveraging existing tools developed for the different model types (see **Table 1**). Cis hopes to uncover general strategies for tool and model integration and grasp where new tool development is necessary.

#### Data Visualization

In the era of “Big Data,” a key challenge faced by the plant sciences community is effective visualization of large experimental or simulation datasets to reveal hidden insights. The Cis interface will provide access to model integration tools and enable visualization of model outputs as graphs, tables, and animations. It is anticipated that interactive visualizations of integrated model outputs will intuitively convey simulation dynamics and reveal emergent behaviors that will help researchers to identify new questions. With the proposed modular framework, it will be possible to integrate current, open-source visualization tools into the Cis platform, such as Houdini^14^ and L-Studio (Prusinkiewicz, 2004). High-quality visualizations of the results from integrated and multi-scale modeling will be valuable not only to domain experts, but also to inform producers, farmers, breeders, and the broad public. This transition from investigator-based interactive visualization to end-user and public-based presentation visualization can increase the transparency of scientific research and make it understandable to a broad audience.

### Sustainability

Robert Burns wrote that the best laid schemes of mice and men often go awry (English translation). This phrase can be applied to many well-meaning attempts to create biological tools designed to make the lives of scientists easier or our data more meaningful. However, without community buy-in and nurturing, the best laid schemes often fall by the wayside. At the outset of Crops *in silico* it is critical to devote thought to sustaining this platform into the future, through both financial support and cyberinfrastructure. Aside from traditional sources for funding (federal and private), one option for financial support is to form a not-for-profit association dedicated to sustaining the Cis platform. This route has been successfully followed by the OpenMI platform, which was established in 2007 and sustained through today. Key to the long-term success of Cis is frequent and open conversations among stakeholders to encourage usage. This will be facilitated through the annual meetings described earlier, but also by hosting workshops and other convenings to introduce, teach, and improve the Cis framework and software. The Cis framework must also maintain and expand its user base by including thorough documentation and tutorials that are easy to follow. To be truly user-friendly, it must have an intuitive and easy-to-navigate interface; effective error handling; and, at the very least, work (i.e., have a robust underlying structure). This idealized tool can come to fruition by including computer scientists, information technologists, and graphic artists in the Cis community.

## Conclusion

Famous biological models such as the Lotka–Volterra predator-prey models (Lotka, 1920; Volterra, 1931), the Farquhar, von Caemmerer, and Berry model of photosynthesis (Farquhar et al., 1980), and the Hodgkin–Huxley membrane potential models (Hodgkin and Huxley, 1952) have provided the scientific community with unprecedented understanding of biological processes through simulations of unknown states. These and other models demonstrate the kinds of insights that are only achievable through modeling. We are at a point in history where we have both the need and the capability to use information and models at multiple levels to model whole systems and to achieve greater insights into how whole plants and ecosystems will respond to genetic changes, as well as environmental challenges never before encountered. The Crops *in silico* initiative has the potential to be a powerful discovery tool in which dozens of simulations across multiple scenarios can be accomplished in a few hours. It will be the first framework to enable customized integration of coherent subsets of existing plant models to address specific biological questions. The incorporation of an intuitive user interface with advanced visualization of integrated model outputs also makes the Cis framework unique. It is evident that many technical and social challenges in the development of Cis remain and will arise. However, with open communication and support of the scientific community across domains of expertise, this improbable vision can become a reality.

## Author Contributions

All authors listed, have made substantial, direct and intellectual contribution to the work, and approved it for publication.

## Conflict of Interest Statement

The authors declare that the research was conducted in the absence of any commercial or financial relationships that could be construed as a potential conflict of interest.

## Footnotes

1 http://cropsinsilico.org

2 https://rd-alliance.org/groups/wheat-data-interoperability-wg.html

3 http://co.mbine.org/

4 http://algorithmicbotany.org/virtual_laboratory/

5 http://www.cs.ox.ac.uk/chaste/

6 https://www.cellml.org/

7 http://sbml.org/Main_Page

8 http://amqp.org

9 http://open-mpi.org

10 http://swift-lang.org

11 http://models.pps.wur.nl/node/943

12 https://data.agmip.org/

13 https://www.betydb.org/

14 https://www.sidefx.com/
